# Green spectrophotometric methods for the determination of metformin–curcumin combination: ICH validation and preliminary bioanalytical applications

**DOI:** 10.1038/s41598-026-67101-z

**Published:** 2026-08-25

**Authors:** Amal A. El-Masry, Dina T. El-Sherbiny, Nora A. Abdallah

**Affiliations:** 1 Department of Medicinal Chemistry, Faculty of Pharmacy, Mansoura University, Mansoura, 35516 Egypt; 2 Pharmaceutical Chemistry Department, Faculty of Pharmacy, Nile Valley University Egypt, Fayoum, 63518 Egypt; 3 Department of Pharmaceutical Analytical Chemistry, Faculty of Pharmacy, Mansoura University, Mansoura, 35516 Egypt

**Keywords:** Derivative spectrophotometry, Ratio subtraction, Metformin, Curcumin, Pharmaceutical dosage forms, Human plasma, Green analysis, Biochemistry, Chemistry

## Abstract

The combined therapeutic use of Metformin and Curcumin has attracted growing interest in diabetes management, owing to metformin’s glucose-lowering effect and curcumin’s antioxidant and anti-inflammatory activities. This clinical relevance necessitates reliable analytical methods for their simultaneous determination. In this study, three simple, selective, and cost-effective complementary spectrophotometric methods were developed and validated for the analysis of metformin and curcumin in dosage forms and human plasma. A zero-order spectrophotometric method enabled the selective determination of curcumin, while a first-derivative method allowed simultaneous quantification of both analytes with improved spectral resolution. A ratio subtraction method was also established for selective metformin determination in the presence of curcumin, effectively resolving spectral overlap without prior separation. All methods exhibited excellent linearity over the concentration range of 0.5–20.0 µg mL^− 1^ (*r* ≥ 0.999). The methods also demonstrated high analytical sensitivity, with limits of detection and quantitation ranging from 0.08 to 0.15 µg mL^− 1^ and 0.23 to 0.45 µg mL^− 1^, respectively. Validation following ICH guidelines confirmed satisfactory accuracy, precision, and selectivity. Statistical comparison with reference methods using Student’s t-test and F-test showed no significant differences at the 95% confidence level. The proposed methods were successfully applied to the determination of both drugs in raw materials, metformin pharmaceutical formulations spiked with curcumin, and spiked human plasma, demonstrating excellent accuracy and precision, with recoveries ranging from 98.73% to 101.06% and RSD values not exceeding 1.50%. Greenness assessment demonstrated environmental compatibility, supporting their use as sustainable analytical tools for routine quality control and bioanalytical applications.

**Supplementary Information:**

The online version contains supplementary material available at 10.1038/s41598-026-67101-z.

## Introduction

Diabetes mellitus is a chronic metabolic disorder characterized by persistent hyperglycemia resulting from impaired insulin secretion, insulin resistance, or both, which ultimately leads to serious microvascular and macrovascular complications. Metformin hydrochloride (MET, Fig. S1a), a biguanide antihyperglycemic agent, is widely prescribed as first-line therapy for type 2 diabetes due to its effectiveness in lowering blood glucose^1^, favorable safety profile, and additional antioxidant and anti-inflammatory properties that may mitigate diabetes-induced oxidative stress and inflammation^2^.

Curcumin (CUR, Fig. S1b), a polyphenolic bioactive compound extracted from the rhizome of *Curcuma longa*, has been extensively studied for its potent antioxidant, anti-inflammatory, and antiglycation effects^3,4^. Mechanistic investigations reveal that CUR modulates multiple signaling pathways related to diabetes and its complications, including AGE-RAGE, PI3K-Akt, and TNF signaling pathways^5^, supporting its potential therapeutic utility in ameliorating metabolic dysregulation and inflammatory responses in diabetes^6^. Clinical and preclinical studies have observed that CUR supplementation can improve insulin sensitivity, reduce oxidative stress biomarkers, and positively influence glycemic control in patients with type 2 diabetes and prediabetic conditions^7^.

Emerging evidence suggests that combining curcumin (CUR) with metformin (MET) provides additive or synergistic therapeutic benefits^8^. In experimental studies, this combination has demonstrated superior glycemic control, improved lipid profiles, reduced glycoxidative and oxidative stress, and enhanced paraoxonase activity compared with either agent alone^9,10^. Clinical evidence has also shown improved insulin sensitivity and high-density lipoprotein (HDL) cholesterol levels in women with polycystic ovary syndrome^11^. Additionally, the MET–CUR combination has exhibited protective effects against drug-induced nephrotoxicity^12^, modulation of immune pathways involved in pain^13^, and enhanced anticancer activity through increased chemotherapy efficacy and inhibition of cancer stem cells^14–16^. These findings highlight the broad therapeutic potential of the MET–CUR combination and support the need for reliable analytical methods for its simultaneous determination.

Despite these advantages, the simultaneous analytical determination of MET and CUR remains challenging because of their spectral overlap and differing physicochemical properties. Therefore, the development of validated, simple, sensitive, and cost-effective spectrophotometric methods for their concurrent determination in pharmaceutical formulations and biological matrices is highly desirable for routine quality control and preliminary bioanalytical applications.

Although numerous analytical methods have been reported for the individual determination of CUR or MET, methods for their simultaneous quantification remain limited. CUR has been predominantly analyzed using HPLC with UV or photodiode array detection in bulk drugs, pharmaceutical formulations, and biological samples, with some methods utilizing fluorescence detection to enhance sensitivity^17–20^. MET has been quantified through UV–Vis spectrophotometry, potentiometry, spectrofluorimetry, as well as chromatographic techniques such as UHPLC and HPLC in tablets and plasma^21–24^. CUR has also been assayed using conventional UV–visible spectrophotometric methods^25,26^. Recently, two reverse-phase HPLC methods were developed and validated for the simultaneous quantification of MET and CUR in pharmaceutical formulations^27,28^. However, these reported methods were limited to pharmaceutical formulations and were not applied to biological matrices. This highlights the need for simple, sensitive, cost-effective, and environmentally friendly analytical methods for the concurrent determination of MET and CUR in pharmaceutical formulations, while also exploring their applicability to complex biological matrices.

Spectrophotometric techniques are widely used for drug analysis due to their simplicity, cost-effectiveness, and rapidity^29–31^. First-derivative spectrophotometry^25^ allows the resolution of overlapping spectra, enabling simultaneous determination of multiple compounds. The ratio subtraction method^32^ is another effective spectrophotometric approach, which eliminates spectral interference from one component to selectively quantify another in mixtures. Together, these methods provide reliable and sensitive options for the analysis of drug combinations such as MET and CUR.

In recent years, increasing emphasis has been placed on the application of green analytical chemistry principles in pharmaceutical analysis to minimize environmental impact while maintaining analytical performance. Considering the greenness of an analytical method during its development is now regarded as an essential aspect of modern method design, particularly in terms of reducing hazardous reagents, solvent consumption, and energy usage. The growing emphasis on green analytical chemistry necessitates the evaluation of analytical methods using standardized greenness assessment tools. Therefore, multiple metrics, including ComplexMoGAPI^33^, BAGI^34^, and AGREE^35^, were employed to comprehensively assess the environmental impact of the proposed spectrophotometric methods. In addition, the multi-color assessment (MA) approach was applied to provide a visual and integrative representation of the greenness profile across different evaluation criteria^36^. Spectrophotometric methods offer significant advantages in green analytical chemistry due to their low reagent consumption, minimal waste generation, reduced energy requirements, and avoidance of hazardous solvents, making them environmentally friendly analytical techniques. Consequently, spectrophotometric methods achieve high greenness scores across various green analytical assessment tools, reflecting their reasonable environmental sustainability and compliance with the principles of green analytical chemistry.

## Experimental

### Apparatus

Spectral measurements were obtained using a Shimadzu UV-1601 PC (Kyoto, Japan) double-beam UV–visible spectrophotometer fitted with matched 1 cm quartz cells. Spectra were smoothed at a wavelength interval (Δλ) of 10 nm, while first-derivative spectra were recorded using a Δλ value of 4 nm. Sample sonication was performed using an ultrasonic bath (Elma Sonic S 100(H), Germany). Mixing was achieved using a vortex mixer (ZX3, Velp Scientific, Italy), and centrifugation was carried out using a Sigma 2-16P centrifuge (Germany).

### Materials and chemicals

Analytical-grade reagents were used throughout the study. MET was of purity of 101.24% and CUR was of purity 99.95% as certified. They were supplied from Minapharm Pharmaceuticals and BIOVEA, Egypt, respectively. Ethanol (product of Sigma Aldrich, Germany) was used. Glucophage^®^ tablets, Batch no. sabEEJ362, was product of Minapharm for Pharmaceuticals and Chemical Industries (Cairo, Egypt), under License of Merck Serono. Human plasma specimens used in this study were obtained from the Blood Bank of Mansoura University Hospital, Egypt. The samples were not collected directly from human participants by the authors. All plasma experiments were performed using plasma obtained from a single donor. Upon receipt, they were preserved at − 80 °C until required for analysis.

### Stock solutions

Primary stock solutions of CUR and MET (100.0 µg/mL) were prepared in ethanol. Subsequent working dilutions were made using the same solvent.

### General procedures

#### Construction of calibration curves

Aliquots equivalent to 0.5–20 µg/mL of each drug were separately transferred from their respective standard solutions into two sets of 10 mL volumetric flasks and diluted to volume with ethanol. The prepared solutions were scanned over the wavelength range of 200.0–600.0 nm against ethanol as a blank. Their zero-order absorption spectra were recorded and stored electronically.

For Method I, the absorbance of the prepared CUR solutions was recorded at 440 nm and correlated with their respective final concentrations to generate a calibration curve. The corresponding regression equation was subsequently derived.

For Method II, the recorded zero-order spectra of both drugs were transformed into first-derivative (D¹) spectra using a scaling factor of 10.0 and a Δλ of 4.0 nm. The peak amplitudes were measured at 245 nm (the zero-crossing point of CUR) for MET and at 467 nm (the zero-crossing point of MET) for CUR. The derivative peak amplitudes were then plotted against the corresponding final concentrations of each drug to construct calibration curves, and the associated regression equations were calculated.

For Method III, MET was quantified by eliminating the influence of CUR. This was achieved by dividing the spectra of MET and laboratory-prepared mixtures by the spectrum of 10 µg mL^− 1^ CUR. The resulting ratio spectra were then processed by subtracting the constant value, followed by multiplication with the same CUR divisor (10 µg mL^− 1^) to reconstruct the zero-order spectra of MET. Finally, the absorbance at 245 nm of these reconstructed spectra was plotted against the corresponding MET concentrations, and the regression equations were calculated.

#### Analysis of laboratory-prepared mixtures

Aliquots of MET and CUR were transferred from their standard solutions into 10 mL volumetric flasks and diluted with ethanol to obtain mixtures of varying ratios. The solutions were scanned over 200–600 nm, and the spectra were stored digitally. Calibration procedures described in   “Construction of calibration curves” were applied to these spectra, and the corresponding regression equations were constructed.

#### Analysis of MET in pharmaceutical dosage form in the presence of spiked CUR

Ten Glucophage^®^ tablets were accurately weighed, powdered, and an amount equivalent to 10 mg MET was dissolved in 50 mL ethanol using 30 min of sonication. Insoluble excipients were removed *via* filtration. Aliquots of the filtrate were transferred into a series of 10 mL volumetric flasks. Appropriate volumes of the CUR standard solution were spiked to obtain mixtures with MET ratios of 1:1, 1:2, 2:1, 1:10, 10:1. The mixtures were then diluted to the volume with ethanol. Each mixture was scanned from 200 to 600 nm, and the spectra were stored. Calibration and quantification were performed following “Construction of calibration curves”, and drug content was determined using the corresponding regression equations.

#### Analysis of studied drugs in spiked human plasma

After gentle thawing of the frozen plasma, 1 mL of human plasma was transferred into 10 mL centrifuge tubes and spiked with varying volumes of MET and CUR stock solutions. The mixtures were vortexed for 1 min, then adjusted to 5 mL with ethanol to precipitate plasma proteins^25,32,37^. After vortexing for 30 s, the samples were centrifuged at 3800 rpm for 30 min. The supernatant was filtered through a 0.45 μm syringe filter, and 1 mL was diluted to 10 mL with ethanol. Blank plasma samples were processed under the same conditions without drug addition. All solutions were scanned from 200 to 600 nm, and the spectra were stored digitally. Quantification was performed using the procedures in “Construction of calibration curves”, and concentrations were calculated from the corresponding regression equations.

## Results and discussion

**Fig. 1:**
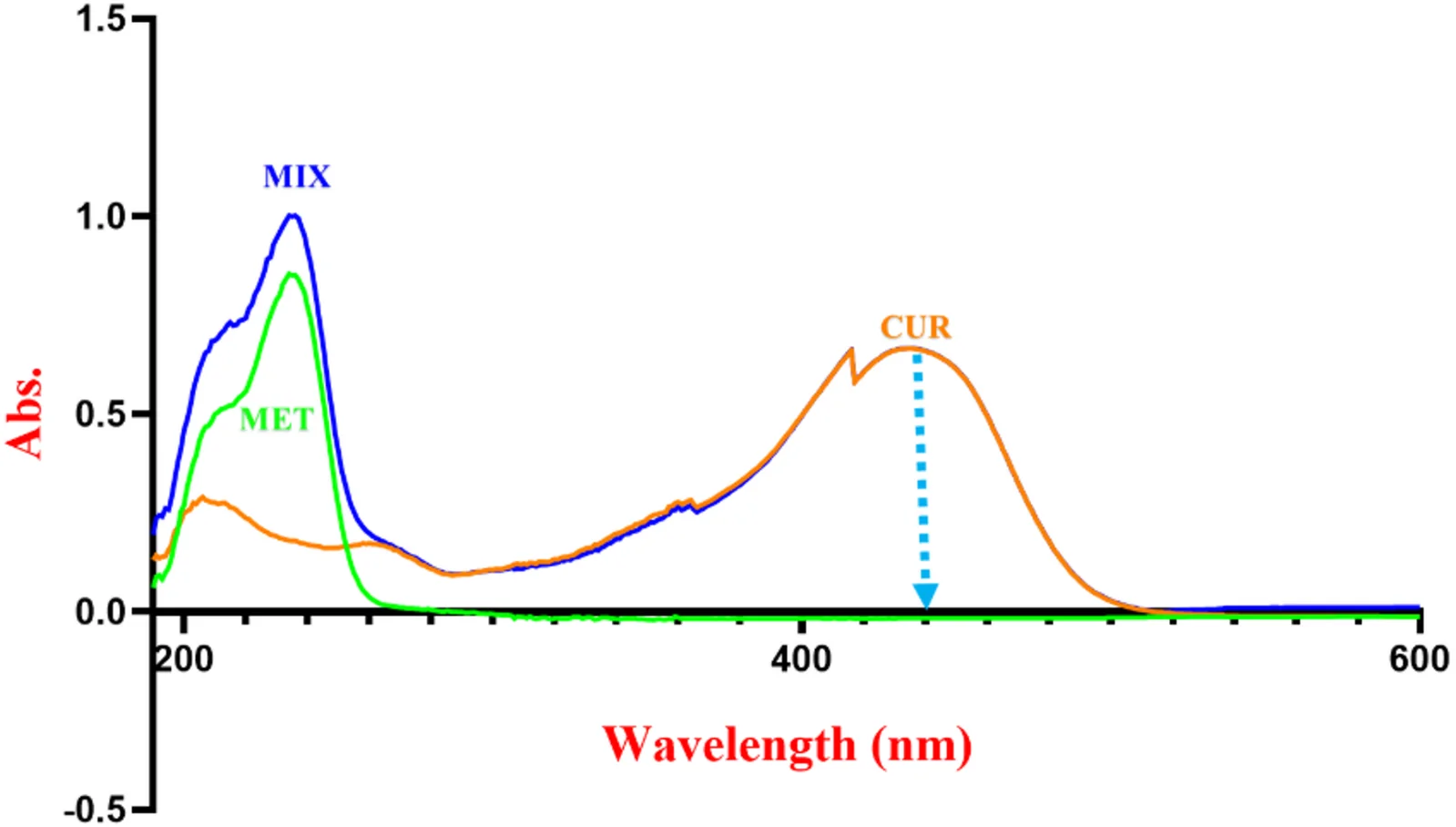
Zero-order absorption spectra of 10.0 µg mL^− 1^ MET, 10.0 µg mL^− 1^ CUR, and their mixture. CUR was measured at 440 nm.

MET and CUR were recently co-administered for diabetes management, combining the glucose-lowering effect of MET with the antioxidant and anti-inflammatory properties of CUR^38,39^. Although two RP-HPLC methods have been reported for the simultaneous determination of MET and CUR, no simple UV spectrophotometric method has yet been reported. Therefore, reliable and efficient spectrophotometric approaches were developed for their combined analysis. The zero-order absorption spectra of MET, CUR, and their synthetic mixture (Fig. 1) showed considerable spectral overlap over most of the UV region. While CUR could be directly determined at 440 nm due to the negligible absorbance of MET, spectral resolution strategies were required for the simultaneous determination of both analytes. Accordingly, three complementary univariate spectrophotometric methods were developed and successfully applied to resolve this binary mixture without prior separation. These approaches proved to be simple, sensitive, cost-effective, and environmentally friendly, allowing accurate quantification of each drug in both pharmaceutical formulations and spiked human plasma. The spectral features observed facilitated the selection of appropriate wavelengths and derivative or ratio manipulations, ensuring reliable calibration and precise assay performance.

### Method I: direct spectrophotometry

Zero-order spectrophotometry remains one of the most straightforward and widely applied quantitative analytical techniques due to its minimal mathematical manipulation and direct relationship between absorbance and concentration according to Beer–Lambert’s law^40^. Its operational simplicity, rapid data acquisition, and suitability for routine quality control make it particularly advantageous when adequate spectral selectivity can be achieved without prior separation^41^. The zero-order absorption spectra of CUR showed a well-defined maximum at 440 nm (Fig. S2), which was utilized for its direct determination. At this wavelength, MET exhibited negligible absorbance, enabling selective quantification without interference. Calibration plots constructed from the recorded absorbance values displayed excellent linearity within the studied concentration range, while regression analysis confirmed the method’s sensitivity and precision. The corresponding regression equations and statistical validation parameters are summarized in Table 1. Furthermore, the applicability of the proposed method to spiked human plasma was demonstrated by the close agreement between the zero-order absorption spectrum of CUR in the spiked plasma mixture containing MET and that of CUR alone at the corresponding concentration (Fig. S3).

### Method II: first-derivative spectrophotometry

**Fig. 2:**
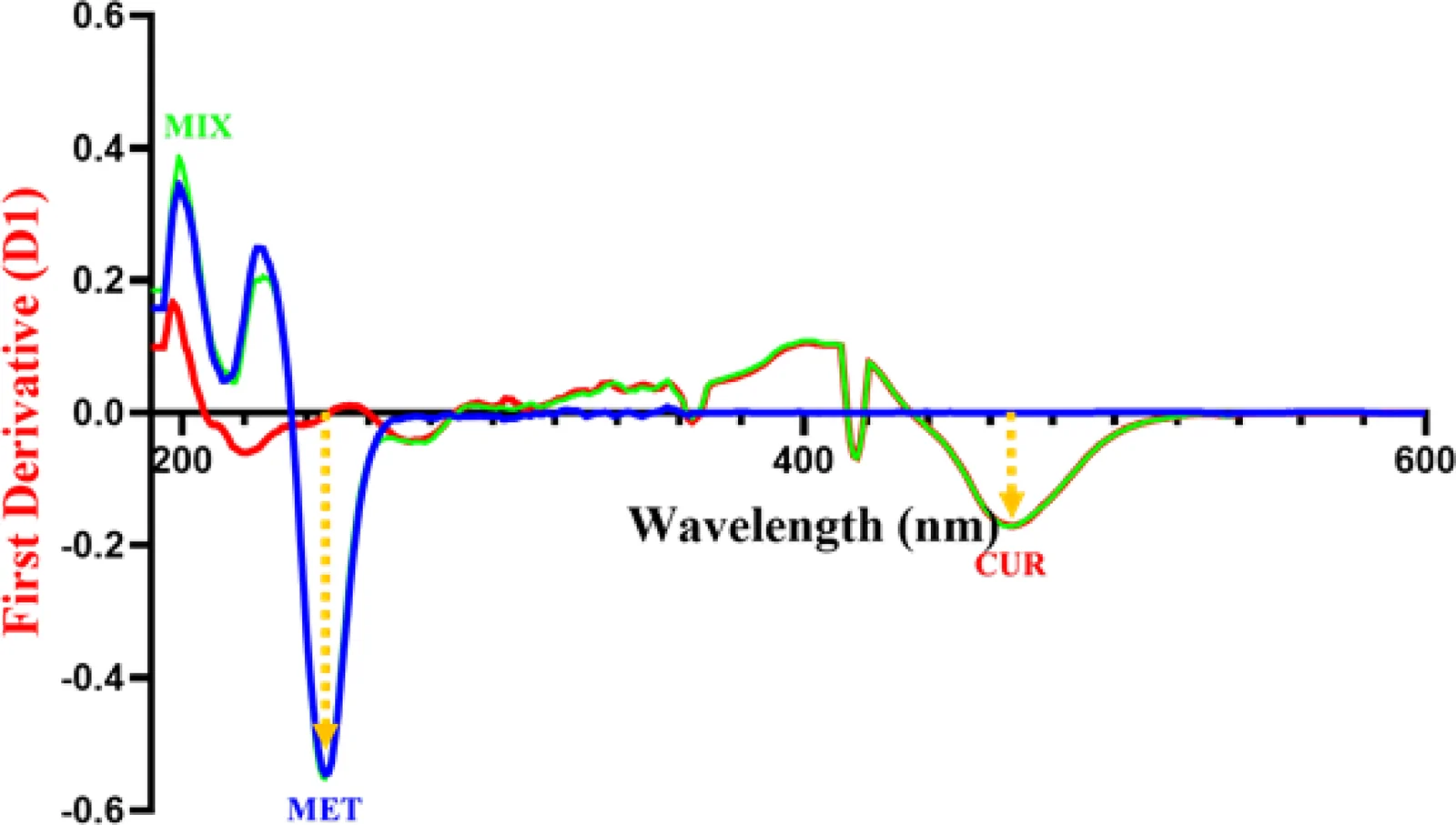
First-derivative spectra of 10.0 µg mL^− 1^ MET) 10.0 µg mL^− 1^ CUR, and their mixture. MET was measured at 245 nm, while CUR was measured at 467 nm.

Derivative spectrophotometry represents a powerful analytical tool for resolving overlapping absorption spectra by computing the first or higher-order derivatives of absorbance as a function of wavelength^42^. This mathematical transformation enhances spectral discrimination, thereby improving the accuracy and selectivity of quantitative analysis in multicomponent systems^25,31,43^. In the present work, the stored zero-order spectra of both drugs were mathematically converted into their first-derivative (D¹) forms. Quantification was achieved by measuring the derivative signal of MET at 245 nm, which corresponds to the zero-crossing point of CUR, while CUR was determined at 467 nm, the zero-crossing point of MET (Fig. 2). This strategy effectively eliminated mutual spectral interference between the two components. The influence of instrumental variables governing derivative signal quality—namely the wavelength increment (Δλ) and scaling factor—was carefully investigated^44^. Various parameter settings were examined to ensure optimal peak definition, signal-to-noise ratio, and analytical sensitivity. A Δλ value of 4.0 nm combined with a scaling factor of 10.0 provided the most favorable balance between resolution and noise suppression. Subsequently, calibration graphs were established by correlating the measured first-derivative amplitudes (D¹) with the respective final concentrations of each analyte (Fig. S4, S5). The corresponding regression equations and statistical performance characteristics are presented in Table 1. Furthermore, the applicability of the first-derivative method to spiked human plasma was demonstrated by the close agreement between the first-derivative spectrum of CUR-MET mixture in the spiked plasma and those of the individual analytes at the corresponding concentrations (Fig. S6).

### Method III: ratio subtraction method (RSM)

**Fig. 3:**
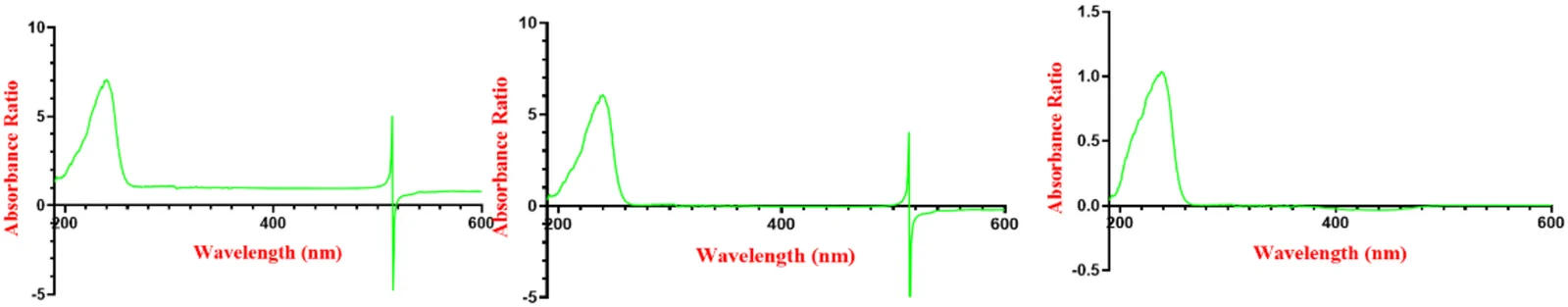
Ratio subtraction method (RSM) applied to MET using CUR (10.0 µg mL^− 1^) as the divisor; the MET spectrum was divided by the divisor, the constant was subtracted, and the resulting spectrum was multiplied by the divisor.

**Fig. 4:**
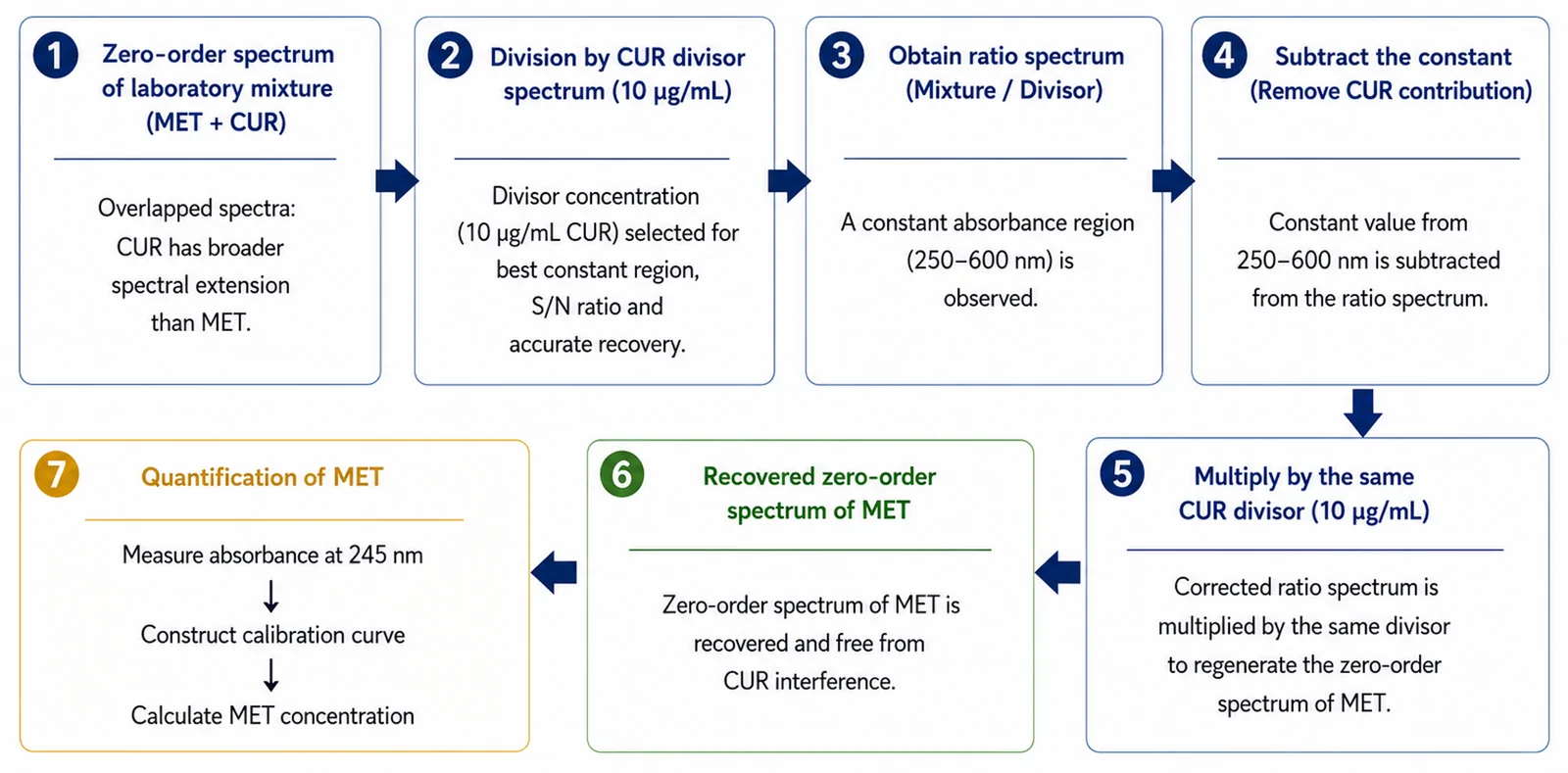
Schematic workflow of the ratio subtraction method (RSM) for the determination of MET in the presence of CUR.

**Fig. 5:**
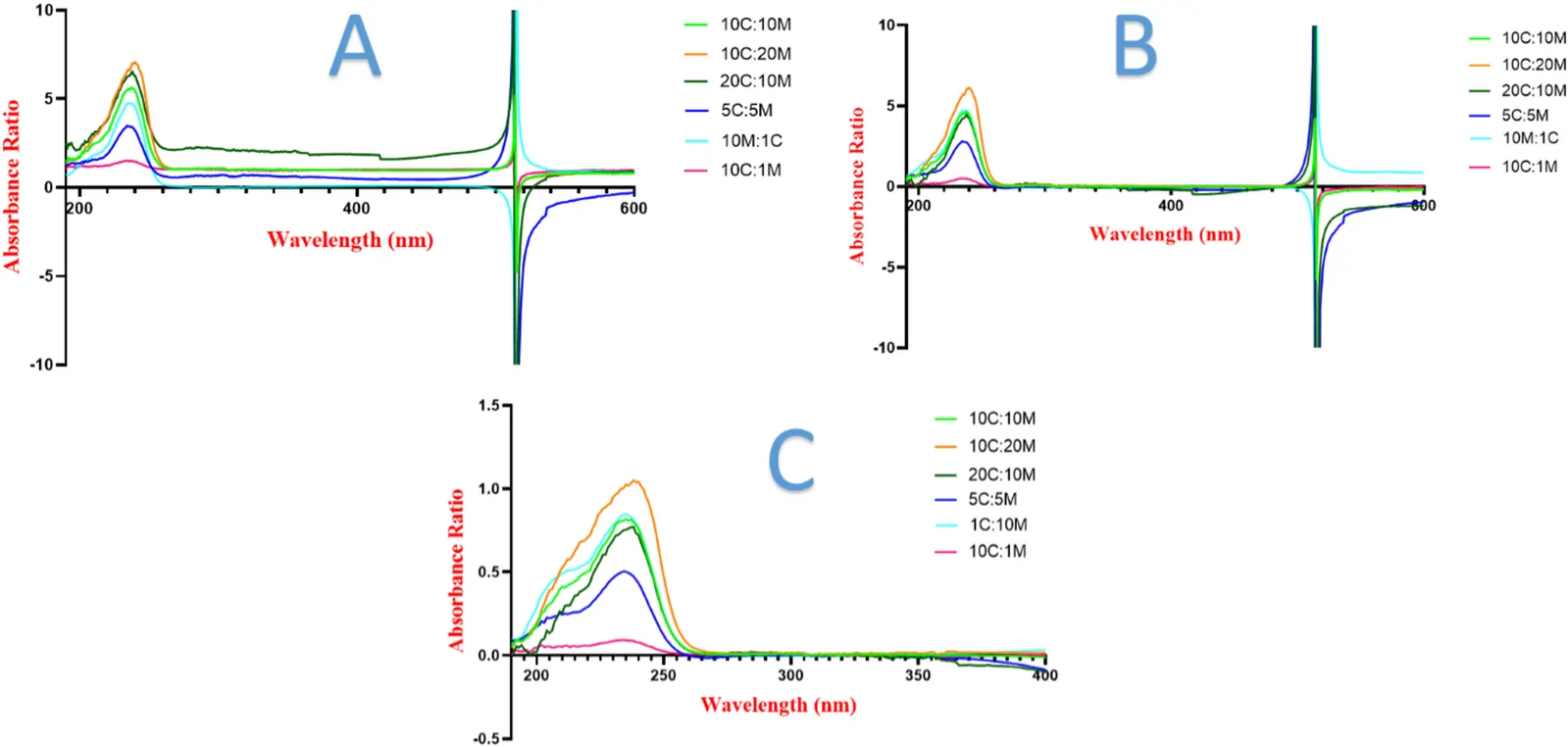
Ratio subtraction method (RSM) applied to synthetic mixtures of MET using CUR (10.0 µg mL^− 1^) as the divisor; the MET spectra were divided by the divisor, the constant was subtracted, and the resulting spectra were multiplied by the divisor.

The absorption subtraction technique, originally described by El-Bardicy et al.^45^, is particularly suitable for binary mixtures exhibiting overlapping spectra where one component shows a more extended zero-order (D⁰) spectrum than the other. In the present case, CUR (A) displays a broader spectral extension compared to MET (B), as illustrated in Fig. 1. Accordingly, CUR was directly quantified by measuring its D⁰ absorbance at 440 nm, a wavelength at which MET shows no significant contribution. A calibration graph was constructed by plotting absorbance values against the corresponding CUR concentrations, and the related regression equation is presented in Table 1. Quantification of MET was achieved by eliminating the spectral contribution of CUR using ratio spectrum manipulation. The zero-order spectra of MET and laboratory-prepared mixtures were divided by the spectrum of CUR (10 µg mL^− 1^) to generate ratio spectra. Different CUR concentrations were evaluated as divisor spectra during method development. A divisor concentration of 10 µg mL^− 1^ was selected because it provided the most reproducible constant region, satisfactory signal-to-noise ratio, and accurate recovery values over the studied concentration range. The constant absorbance region (250–600 nm) was identified and its value subtracted from the ratio spectra to remove CUR interference. The corrected spectra were then multiplied by the same divisor (10 µg mL^− 1^ of CUR) to regenerate the zero-order spectrum of MET (Fig. 3). The absorbance of the reconstructed MET spectra was measured at 245 nm and plotted against the corresponding concentrations to establish the calibration curve. The calculated regression equation is summarized in Table 1. Figure 4 presents a schematic workflow of the ratio subtraction method used for the recovery and determination of MET in the presence of CUR. Figure 5 illustrates the recovered zero-order spectra of MET in laboratory-prepared mixtures after applying RSM. Fig. S7 illustrates the recovered zero-order absorption spectra of MET after applying RSM at different concentrations. The recovered spectra exhibit excellent linearity over the investigated concentration range, confirming the effective elimination of CUR interference without introducing significant spectral distortion or noise amplification, thereby demonstrating the suitability of the method for accurate quantitative analysis. Fig. S8 and S9 illustrates the application of RSM for the analysis of Glucophage^®^ dosage form containing MET, and for the determination of MET in spiked human plasma, respectively.

## Validation

The accuracy, precision, and overall reliability of the proposed methods were systematically evaluated, with validation performed following the International Council for Harmonization (ICH) guidelines^46^ to ensure compliance with regulatory standards.

###  Linearity and range

Calibration curves were constructed for all methods by plotting the analytical response against the corresponding drug concentrations. The results demonstrated good linearity over the concentration range of 0.5–20.0 µg mL^− 1^ for both MET and CUR. Relevant statistical parameters confirming linearity are presented in Table 1, Fig. S10.

### Limit of detection and quantitation

The sensitivity of each proposed method was evaluated by calculating the limit of detection (LOD) and limit of quantitation (LOQ) in accordance with ICH guidelines. LOD and LOQ were derived from the calibration curve using the following relationships:

$${\rm LOD}\,=\,3.3\,{\rm Sa /b \quad \quad \quad LOQ}\,=\,10\,{\rm Sa /b}.$$

Where Sa = standard deviation of the intercept of the calibration curve & b = slope of the calibration curve.

The calculated values for each method are presented in Table 1, confirming their high sensitivity for the simultaneous determination of MET and CUR.

### Accuracy and precision

The analytical performance of the proposed methods was evaluated through recovery studies using the pure drug materials. The results, summarized in Table 2, demonstrated excellent accuracy, with recoveries close to 100% and minimal standard deviations. To further assess method reliability, statistical comparison with previously reported methods^47,48^ was performed using Student’s t-test and the F-test^49^, confirming no significant differences in either accuracy or precision.

Precision was assessed at two levels: intra-day and inter-day. Intra-day precision was determined by analyzing three different concentrations of each drug (2.5, 5.0, and 10.0 µg mL^− 1^) in triplicate within a single day, while inter-day precision was evaluated by repeating the measurements across three consecutive days. The low percent relative standard deviation (%RSD) values obtained (Table 3) indicate the high reproducibility of the proposed methods for simultaneous quantification of MET and CUR.

### Selectivity

The selectivity of the proposed methods was rigorously demonstrated by analyzing MET and CUR in laboratory-prepared mixtures, commercial dosage forms, and spiked human plasma. The excellent recovery results presented in Tables 4 and 5 confirm that neither excipients nor plasma components interfered with the measurements, and that MET and CUR did not influence each other’s quantification. These findings underscore the specificity of the methods, highlighting their suitability for precise simultaneous determination of both drugs in complex matrices.

## Applications

### Analysis of MET and CUR in synthetic mixtures and dosage forms

The proposed methods successfully enabled the simultaneous determination of MET and CUR in laboratory-prepared mixtures with varying drug ratios. They were also successfully applied to MET pharmaceutical formulations spiked with CUR, supporting their application for the analysis of co-administered MET and CUR. The results demonstrated excellent accuracy and precision, with recovery values close to 100%, as summarized in Table 5, highlighting the potential for the simultaneous determination of co-administered MET and CUR in routine quality control applications.

**Table 1:** Analytical data for the assay of MET and CUR through the laboratory performed different spectrophotometric methods.

Methods	^1^D	RSM	^0^D	^1^D
Parameter	MET		CUR	
	245 nm		440 nm	467 nm
Conc. range (µg mL^− 1^)	0.5–20.0			
Correlation coefficient	0.999			
Regression equation	0.004 + 0.043 C	0.05 + 0.058 C	0.02 + 0.05 C	0.09 + 0.02 C
Slope (b)	0.043	0.058	0.05	0.02
Intercept	0.004	0.05	0.02	0.09
LOD^a^ (µg mL^− 1^)	0.10	0.15	0.08	0.1
LOQ^b^ (µg mL^− 1^)	0.31	0.45	0.23	0.3
S_y/x_^c^	24 × 10^− 4^	46 × 10^− 4^	22 × 10^− 4^	8 × 10^− 5^
S_a_^d^	13 × 10^− 4^	26 × 10^− 4^	12 × 10^− 4^	46 × 10^− 5^
S_b_^e^	13 × 10^− 5^	25 × 10^− 5^	12 × 10^− 5^	4 × 10^− 5^
% RSD^f^	1.16	1.37	1.16	1.26
% Er^g^	0.43	0.52	0.44	0.48

^a^Detection limit, ^b^ Quantitation limit, ^c^ Standard deviation of residuals, ^d^ Standard deviation of intercept, ^e^ Standard deviation of slope, ^f^ Percentage of relative standard deviation, ^g^ Percentage of relative error.

**Table 2:** Analytical data for the assay of MET and CUR in raw material by the investigated different spectrophotometric methods and the other comparison ones.

Parameter	Proposed methods				Comparison methods	
	% Found^a^					
	^1^D	RSM	^0^D	^1^D	^45,46^	
Taken concentration (µg mL^− 1^)	MET		CUR		MET	CUR
	245 nm		440 nm	467 nm		
0.5	98.40	99.40	98.20	98.80	100.20	97.92
1.0	98.10	98.10	98.30	98.10	102.76	100.94
2.5	99.88	101.84	98.80	98.16	101.9	100.38
5.0	101.02	98.76	101.28	101.66	100.09	99.81
10.0	101.09	101.53	100.45	100.19	-	-
15.0	100.03	99.81	99.80	99.70	-	-
20.0	99.97	99.82	99.95	99.78	-	-
$\bar{\rm X}$^a^ ± SD	99.78 ± 1.16	99.89 ± 1.37	99.54 ± 1.15	99.48 ± 1.26	101.24 ± 1.30	99.76 ± 1.31
*t-*value	1.91 (2.26)	1.59 (2.26)	0.29 (2.26)	0.35 (2.26)		
F-value	1.27 (9.28)	1.09 (9.28)	1.3 (9.28)	1.1 (9.28)		

^a^Each result is the mean recovery of three individual analyses.

The values between brackets are tabulated *t-* and *F*-values at *P* = 0.05 (95% confidence level), with degree of freedom (df) (df = 9 for *t-*value and df_1_ = 6, df_2_ = 3 for F-value).

**Table 3:** Precision data for analysis of MET and CUR by the spectrophotometric methods.

Methods			^1^D					RSM				
Drug			MET concentrations (µg.mL^− 1^)									
Parameters			2.5	5.0		10.0		2.5		5.0		10.0
Intra-day		$\bar{\rm X}$ ^a^	100.62	100.87		100.08		101.14		99.33		101.37
		± SD	0.38	0.42		0.24		0.69		0.53		0.30
		% RSD	0.37	0.41		0.24		0.68		0.53		0.30
		% Error	0.22	0.24		0.14		0.39		0.31		0.17
Inter-day		$\bar{\rm X}$ ^b^	101.31	100.24		100.08		100.68		98.77		101.04
		± SD	0.99	1.28		1.32		1.05		0.71		0.92
		% RSD	0.97	1.28		1.32		1.05		0.71		0.91
		% Error	0.56	0.74		0.76		0.60		0.41		0.52

Methods			^0^D					^1^D				
Drug			CUR concentrations (µg.mL^− 1^)									
Parameters			2.5		5.0		10.0	2.5			5.0	10.0
Intra-day	$\bar{\rm X}$ ^a^		98.28		100.15		100.43	99.56			101.83	99.98
	± SD		0.44		1.37		0.20	1.3			0.34	0.98
	% RSD		0.45		1.36		0.20	1.31			0.34	0.98
	% Error		0.26		0.79		0.12	0.76			0.19	0.57
Inter-day	$\bar{\rm X}$ ^b^		99.65		99.52		100.27	99.45			100.64	100.8
	± SD		0.83		1.53		0.87	1.39			0.93	1.39
	% RSD		0.84		1.54		0.87	1.40			0.92	1.38
	% Error		0.48		0.89		0.5	0.81			0.53	0.80

$\bar{\rm X}$^a^: Mean recovery of triple analyses of the analyte in the same day.

$\bar{\rm X}$^b^: Mean recovery of triple analyses of the analyte in three consecutive days.

**Table 4:** Determination of MET and CUR in laboratory synthetic mixture by the proposed methods.

Ratios		MET %Recovery^a^		CUR %Recovery^a^	
MET: CUR	Concentration (µg mL^− 1^)	^1^D	RSM	^0^D	^1^D
1: 1	5: 5	98.24	98.42	102.42	101.67
1: 1	10: 10	98.77	99.81	98.56	101.47
1: 2	10:20	101.09	101.53	101.93	99.46
2: 1	20: 10	99.27	99.82	100.83	98.91
1: 10	1:10	101.39	103.10	100.45	100.83
10: 1	10:1	98.77	99.91	101.14	98.08
$\bar{\rm X}$^a^ ± SD		99.59 ± 1.32	100.43 ± 1.64	100.89 ± 1.35	100.07 ± 1.47

^a^Each result is the mean recovery of three individual analyses.

**Table 5:** Determination of MET and CUR in dosage form and spiked human plasma by the proposed methods.

Application	Ratios		MET %Recovery^a^		CUR %Recovery^a^	
	MET: CUR	Concentration (µg mL^− 1^)	^1^D	RSM	^0^D	^1^D
Glucophage^®^^b^	1: 1	5: 5	101.48	99.45	101.29	102.31
	1: 1	10: 10	102.02	101.53	100.45	100.83
	1: 2	10: 20	101.79	101.02	102.03	98.17
	2: 1	20: 10	100.43	101.54	100.64	101.47
	1: 10	1: 10	101.39	99.66	100.45	102.12
	10: 1	10: 1	99.23	100.94	100.57	101.28
	$\bar{\rm X}$^a^ ± SD		101.06 ± 1.05	100.69 ± 0.92	100.91 ± 0.63	101.03 ± 1.5
Spiked plasma	1: 1	0.5: 0.5	98.53	98.01	99.76	99.99
	1: 2	0.5: 1	99.13	97.87	100.54	100.38
	2: 1	1: 0.5	99.65	99.63	99.52	98.81
	10: 1	5: 0.5	100.92	99.95	98.59	99.07
	1: 10	0.5: 5	98.03	98.19	101.54	101.95
	$\bar{\rm X}$^a^ ± SD		99.25 ± 1.11	98.73 ± 0.98	99.99 ± 1.06	100.04 ± 1.25

^a^Each result is the mean recovery of three individual analyses.

^b^Glucophage^®^ tablets (500.0 mg), batch no. sabEEJ362, was product of Minapharm for Pharmaceuticals and Chemical Industries (Cairo, Egypt), under License of Merck Serono.

### Analysis of MET and CUR in spiked human plasma

The high sensitivity of the developed methods allowed for the simultaneous quantification of MET and CUR in spiked human plasma. Following administration of a single 1.5 g oral dose of MET, peak plasma concentrations of 1.8–4.0 µg mL^− 1^ were reported after approximately 2 h^50^, while CUR exhibited maximum plasma levels of 0.19–0.60 µg mL^− 1^ after daily doses of 4–8 g^51,52^. When the measured responses were plotted against the corresponding drug concentrations in spiked plasma samples, linear relationships were obtained. The high percent recoveries and low standard deviations (Table 5) demonstrate that the proposed methods provide accurate, precise, and selective determination of MET and CUR in complex biological matrices, highlighting their potential for pharmacokinetic and clinical applications.

###  Greenness assessment of the proposed spectrophotometric methods

**Fig. 6:**
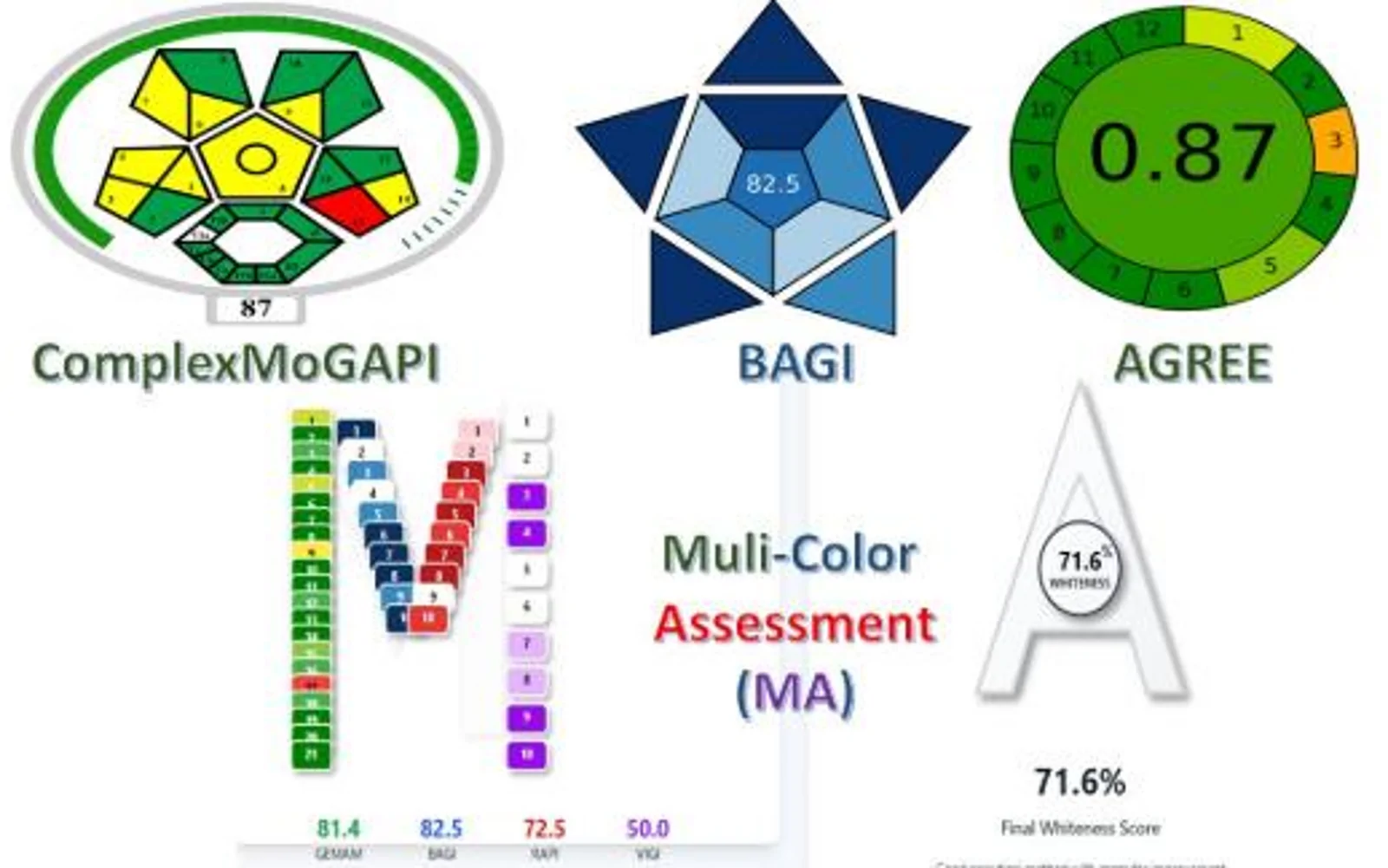
Assessment of the proposed method applying ComplexMoGAPI, BAGI, AGREE, MA tool.

The greenness profile of the proposed spectrophotometric methods was evaluated using four complementary assessment tools, namely ComplexMoGAPI, BAGI, AGREE, and the multi-color assessment (MA), as illustrated in Fig. 6. The ComplexMoGAPI index yielded a score of 87, indicating a high level of compliance with green chemistry principles, particularly in terms of reduced hazardous reagents and minimized waste generation. Similarly, the BAGI assessment demonstrated a strong performance with a score of 82.5, reflecting efficient analytical conditions and low environmental burden. Furthermore, the AGREE metric provided an overall score of 0.87, highlighting excellent alignment with the twelve principles of green analytical chemistry. The multi-color assessment (MA) further supported these findings, revealing a predominance of green zones with limited yellow and minimal red areas, which indicates minor environmental concerns. Additionally, the whiteness score of 71.6% confirms a balanced integration of analytical performance, environmental sustainability, and practical applicability. Collectively, these findings demonstrate that the proposed methods provide an environmentally friendly alternative with satisfactory analytical performance for the routine determination of MET and CUR. Furthermore, the greenness and sustainability of the proposed method for the analysis of spiked human plasma were evaluated using the ComplexMoGAPI, BAGI, and AGREE assessment tools (Fig. S11), confirming its environmentally friendly analytical profile.

### **Comparison of analytical performance and greenness**

Compared with the recently reported RP-HPLC methods^27,28^, the proposed spectrophotometric methods provide a simple, rapid, cost-effective, and environmentally sustainable alternative for the simultaneous determination of MET and CUR. Notably, the proposed methods achieved comparable or improved analytical sensitivity, as reflected by their lower working concentration limits and wider linear ranges (Table S1). Furthermore, unlike the reported RP-HPLC methods, which were applied only to bulk materials and pharmaceutical formulations, the present methods were also successfully demonstrated in spiked human plasma as a proof-of-concept application. A comprehensive comparison of the analytical performance and greenness profiles of the proposed and reported methods is presented in Table S1.

## Conclusion

MET and CUR represent a promising therapeutic combination in diabetes management, offering complementary benefits through glycemic control and the mitigation of oxidative stress and inflammation. Despite this clinical relevance, no UV-spectrophotometric method has previously been reported for their simultaneous determination. In this study, three validated spectrophotometric methods, namely zero-order, first-derivative, and absorption subtraction, were developed for the first time for the concurrent analysis of MET and CUR in pharmaceutical formulations and demonstrated successful proof-of-concept applicability in spiked human plasma. The proposed methods exhibited excellent analytical performance, including high sensitivity, precision, selectivity, wide linear ranges, low detection limits, and satisfactory recoveries. Moreover, comparison with the reported RP-HPLC methods demonstrated comparable or superior analytical sensitivity while offering the advantages of simplicity, rapidity, cost-effectiveness, and environmental compatibility. Although the biological application was limited to spiked human plasma, the encouraging results establish a solid foundation for future investigations involving authentic clinical samples, comprehensive bioanalytical validation, and pharmacokinetic studies of the MET–CUR combination.

## Supplementary Information

Below is the link to the electronic supplementary material.

Supplementary Material 1

## Data Availability

Data will be available upon request.
