# Supplementary material for: Green spectrophotometric methods for the determination of metformin–curcumin combination: ICH validation and preliminary bioanalytical applications
Source: Sci Rep. 2026 Aug 25;16:26686. doi: 10.1038/s41598-026-67101-z (PMC13507117; doi:10.1038/s41598-026-67101-z)
Supplement: Supplementary file 1 [file 41598_2026_67101_MOESM1_ESM.docx]

**Fig. S1. Chemical structure of the studied drugs**

**Fig. S2. Zero-order absorption spectra showing the calibration of CUR in the presence of 10.0 µg/mL MET, measured at 440 nm.**

**Fig. S3. Zero-order absorption spectra demonstrating the determination of 5.0 µg/mL CUR in spiked human plasma in the presence of 0.5 µg/mL MET (blue curve), compared with the spectrum of 5.0 µg/mL CUR alone (pink curve).**

**Fig. S4. First-derivative spectra of CUR at different concentrations in the presence of 10.0 µg/mL MET.**

**Fig. S5. First-derivative spectra of MET at different concentrations in the presence of 10.0 µg/mL CUR.**

**Fig. S6. First-derivative spectra of 0.5 µg/mL MET (blue curve), 5.0 µg/mL CUR (green curve), and their mixture in spiked human plasma (pink curve).**

**Fig. S7. Ratio subtraction method (RSM) applied to MET at different concentrations using CUR (10.0 µg/mL) as the divisor; the MET spectra were divided by the divisor, the constant was subtracted, and the resulting spectra were multiplied by the divisor.**

**Fig. S8. Ratio subtraction method (RSM) applied to Glucophage^®^ dosage form containing MET using CUR (10.0 µg/mL) as the divisor; the MET spectra were divided by the divisor, the constant was subtracted, and the resulting spectra were multiplied by the divisor. Where M: MET, C: CUR.**

**Fig. S9. Ratio subtraction method (RSM) spectra of (1, blue curve) spiked human plasma containing MET (1.0** **µg/mL) and CUR (0.5 µg/mL)), compared with (2, pink curve) a laboratory-prepared mixture of MET and CUR.**

**Fig. S10. Calibration curves for MET and CUR obtained using the proposed spectrophotometric methods. Each data point represents the mean of three replicate measurements, and the error bars indicate the standard deviation (SD).**

**Fig. S11. Greenness and sustainability assessment of the proposed method for spiked human plasma analysis using the ComplexMoGAPI, BAGI, AGREE, and MA assessment tools.**

**Table S1: Comparison of the proposed method with reported HPLC methods using the ComplexMoGAPI, BAGI, and AGREE assessment tools.**

| **The Method** | **Range**  **(µg mL^-1^)** | **LOD/LOQ** | **Analysis time** | **The Greenness Assessment Tools** |
| --- | --- | --- | --- | --- |
| **Proposed Method** | **0.5-20** | **MET:**  **0.10-0.15**  **0.31-0.45**  **CUR:**  **0.08-0.1**  **0.23-0.3** | **1 min.** |  |
| **Reported HPLC**  [1] | **2-10 µg mL^-1^** | **MET:**  **0.30**  **0.91**  **CUR:**  **0.21**  **0.63** | **8 min.** |  |
| **Reported HPLC** [2] | **MET:**  **10-100 µg mL^-1^**  **10-50 µg mL^-1^** | **MET:**  **6.85**  **20.76**  **CUR:**  **3.49**  **10.56** | **6 min.** |  |

**References:**

**1.** Prasanna, B. J. V. L., Raju, G. E., Raghava, D. & Rao, K. N. Method development and validation for simultaneous estimation of curcumin and metformin in bulk and polyherbal formulations by RP-HPLC. Cuestiones de Fisioterapia **54**, 1295–1313 (2025).

**2.** Noshadi, B., Burgaz, E. V. & Pozharani, L. B. Development and validation of an RP-HPLC method for the simultaneous quantification of metformin and curcumin. Cyprus Journal of Medical Sciences (2025). <https://doi.org/10.4274/cjms.2024.2024-132>
